# Hormone replacement therapy in BRCA mutation carriers and risk of ovarian, endometrial, and breast cancer: a systematic review

**DOI:** 10.1007/s00432-021-03629-z

**Published:** 2021-04-22

**Authors:** D. Huber, S. Seitz, K. Kast, G. Emons, O. Ortmann

**Affiliations:** 1 Department of Gynecology and Obstetrics, University Medical Center Regensburg, Regensburg, Germany; 2 Center for Hereditary Breast and Ovarian Cancer, University Hospital of Cologne, Cologne, Germany; 3 Department of Gynecology and Obstetrics, Georg August University Göttingen, University Medicine, Göttingen, Germany

**Keywords:** Hormone replacement therapy, Breast cancer, Ovarian cancer, *BRCA1*, *BRCA2*

## Abstract

**Purpose:**

*BRCA* mutation carriers have an increased risk of developing breast or ovarian cancer. Risk-reducing bilateral salpingo-oophorectomy (RRBSO) is associated with a decrease in risk for tubal and ovarian cancer. Hormone replacement therapy (HRT) may increase breast, ovarian, and endometrial cancer risk in the general population. This review analyses the published data on HRT and risk of cancer in *BRCA* mutation carriers with and without RRBSO.

**Methods:**

We included all relevant articles published in English from 1995 to October 2020. Sources were identified through a search on PubMed and Cochrane Library.

**Results:**

We included one case–control and one retrospective cohort study on ovarian and one case–control study on endometrial cancer risk and HRT in *BRCA* mutation carriers. Regarding breast cancer risk, one case–control study on *BRCA* mutation carriers with and without RRBSO and one case–control study, one Markov chain decision model, two prospective cohort studies, and one metaanalysis on carriers after RRBSO were included. For ovarian cancer, results were ambiguous. For breast cancer, most studies did not find an adverse effect associated with HRT. However, some of the studies found a risk modification associated with different formulations and duration of use.

**Conclusion:**

Although data are limited, HRT does not seem to have a relevant effect on cancer risk in *BRCA* mutation carriers. RRBSO should not be postponed to avoid subsequent HRT in this population. Adequate HRT after RRBSO should be offered to avoid chronic diseases resulting from low estrogen levels. However, further data on the safety of different formulations are needed.

## Introduction

Hormone replacement therapy (HRT) is the most effective treatment for climacteric symptoms. Furthermore, adequate HRT may avoid chronic diseases resulting from low estrogen levels such as osteoporosis and myocardial infarction, especially in younger women with premature ovarian failure. (S^3^-Leitlinie Peri 2020) However, HRT may increase breast, ovarian, and endometrial cancer risk in the general population. The extent of the increase in risk depends on type and duration of HRT.

A recent metaanalysis shows a significant time dependent increase in breast cancer risk associated with HRT. The increase in risk was higher for combined estrogen–progestin therapy (EPT) than for estrogen-only therapy (ET). (Collaborative Group on Hormonal Factors in Breast Cancer 2019) In hysterectomized women, ET is, therefore, recommended, when HRT is needed. Recent metaanalyses also show an increased ovarian cancer risk in HRT users. The observed increase in risk was associated with ET and EPT. Among current users, an increased risk was observed after a duration of less than 5 years. (S3-Leitlinie Peri 2020) Regarding endometrial cancer, ET is associated with an increase in risk in non-hysterectomized women. Short-term EPT up to 5 years of use does not seem to have a significant impact. (Beral et al. 2005) However, long-term EPT with a duration of use of more than 10 years can cause an increase in endometrial cancer risk. (Razavi et al. 2010) Therefore, ET should be used only in hysterectomized women (S3-Leitlinie Peri 2020).

The increase in cancer risk might be especially relevant for high-risk subgroups. According to recent prospective data, the cumulative risk at the age of 80 years in *BRCA1* mutation carriers is up to 72% for breast cancer and up to 44% for ovarian cancer. In *BRCA2* mutation carriers, the cumulative breast cancer risk at the age of 80 years according to these data is increased up to 69% and for ovarian cancer up to 17%, respectively (Kuchenbaecker et al. 2017). In a high-risk subgroup, possible risk modifiers are, therefore, to be used with caution. Several retrospective studies suggested that risk-reducing bilateral salpingo-oophorectomy (RRBSO) is associated with reduction in risk not only for ovarian and fallopian tube cancer, but also for breast cancer in *BRCA1/2* mutation carriers (Rebbeck et al. 2009; Domchek et al. 2010). However, risk reduction for breast cancer after RRBSO in *BRCA1/*2 mutation carriers may have been overestimated because of selection bias in the existing observational studies (Heemskerk-Gerritsen et al. 2015). Prospective studies could not confirm a risk reduction for breast cancer especially in *BRCA1* mutation carriers (Mavaddat et al. 2020). In this study, we analyze the published data on HRT and risk for breast or ovarian cancer in *BRCA1/2* mutation carriers with and without RRBSO.

## Methods

A search for relevant articles was run from January 1995 to October 2020 on PubMed. The MeSH used for the search was: “hormone replacement therapy”, “BRCA mutation”, “hormone replacement therapy”, “breast cancer” or “ovarian cancer”. The search created 70, 4548 and 877 hits, respectively. We also conducted a search within the Cochrane library. There were no Cochrane reviews available on HRT and risk of cancer in *BRCA1/2* mutation carriers. The hits were searched for relevance, clinical trials, reviews, and metaanalyses. The authors of this review defined all articles as relevant which deal with the specific subject of whether or not HRT has an impact on breast and/or ovarian cancer in *BRCA1/2* mutation carriers.

We found 11 relevant publications, 2 of which regarding ovarian cancer, 8 regarding breast cancer and 1 regarding endometrial cancer risk. 6 of the publications which investigated breast cancer risk focused on *BRCA1/*2 mutation carriers who had undergone RRBSO. After excluding crossmatch, there remained a total of nine publications to include in our systematic review. We included one case–control and one retrospective cohort study on HRT and ovarian cancer risk as well as one case–control study on HRT and breast cancer risk in *BRCA1/2* mutation carriers with and without RRBSO. Furthermore, we included one case–control study, one Markov chain decision model, two prospective cohort studies and one metaanaylsis which examined the association between HRT and breast cancer risk in women with *BRCA1/2* who had undergone RRBSO. Also, we included one case–control study on HRT-induced endometrial cancer risk in *BRCA1/2* mutation carriers in our review.

## Results

### Ovarian cancer

We found two adequate studies on the association between HRT and ovarian cancer risk in *BRCA1/2* mutation carriers to include in our review (Table 1).

**Table 1:** HRT and risk of ovarian cancer in BRCA mutation carriers

Study/study design/Oxford center of evidence based medicine (OCEBM) level of evidence (LOE)	Number	Results
Kotsopoulos et al. (2006) Case–control study LOE 3b	Cases: 117 *BRCA1*, 45 *BRCA2* Controls: 256 *BRCA1*, 119 *BRCA2*	No increase in risk overall *BRCA1*: OR = 0.92; 95% CI 0.50–1.70; *P *= 0.80 *BRCA2*: OR = 0.89; 95% CI 0.29–2.39; *P *= 0.74 Non-significant increase in risk associated with ever use of estrogen (OR = 1.5; 95% CI 0.73–3.11), non-significant decrease in risk associated with ever use of progestin (OR = 0.57; 95% CI 0.24–1.35) No association with duration of use
Perri et al. (2015) Retrospective cohort study LOE 2b	718 *BRCA1,* 331 *BRCA2* (use of HRT in total: 105)	Increase in risk *BRCA1*: OR = 1. 66; 95% CI 0.89–3.08; *P *< 0.001 *BRCA2*: OR = 3.04; 95% CI 1.19–7.8; *P *< 0.001

A case–control study by Kotsopoulos et al. from 2006 found no increase in ovarian cancer risk associated with use of HRT in *BRCA1/2* mutation carriers (OR = 0.93; 95% CI 0.56–1.56; *P *= 0.79) (Kotsopoulos et al. 2006). The results were similar when tested separately for *BRCA1* (OR = 0.92; 95% CI 0.50–1.70; *P *= 0.80) and *BRCA2* mutation carriers (OR = 0.89; 95% CI 0.29–2.39; *P *= 0.74). They included 117 cases and 256 controls with *BRCA1* as well as 45 cases and 119 controls with *BRCA2* mutation. All women had intact ovaries and underwent natural menopause. The mean age at enrollment was 62.7 years for cases and 61.2 years for controls (*P *= 0.51). A total of 45 (27.8%) cases and 83 (22.1%) controls reported ever use of HRT (*P *= 0.33). The average duration of HRT use was 5.4 years for cases and 7.6 years for controls, the difference was statistically significant (*P *= 0.05). 31 of the cases and 65 of the controls reported ever use of estrogen-containing HRT, and 19 of the cases and 48 of the controls progestin-based HRT. No significant association was found with ovarian cancer risk and duration of HRT use (*BRCA1*: *P *= 0.14; *BRCA2*: *P *= 0.22). When the type of HRT was examined, the study found a modest increase in ovarian cancer risk associated with ever use of estrogen (OR = 1.5; 95% CI 0.73–3,11) and a decrease in risk associated with ever use of progestin (OR = 0.57; 95% CI 0.24–1.35) compared with never use of HRT. However, both effects were not significant. Use of estrogen was defined as use of either estrogen or estrogen and progestin-based HRT, use of progestin as use of either progestin or estrogen and progestin-based HRT. The effects associated with different formulations of HRT were not investigated separately for *BRCA1* and *BRCA2* mutation carriers.

Perri et al. included a total of 718 *BRCA1* and 331 *BRCA2* mutation carriers to conduct a retrospective cohort study (Perri et al. 2015). The main purpose of the study was to investigate the association between ovarian cancer and fertility treatments. Also, the study investigated potential confounding effects including use of HRT. Multivariate analysis showed an increase in ovarian cancer risk associated with use of HRT for *BRCA1/2* mutation carriers (OR = 1.98; 95% CI 1.21–3.25). Analysis was performed separately for *BRCA1* (OR = 1.66; 95% CI 0.89–3.08; *P *< 0.001) and *BRCA2* (OR = 3.04; 95% CI 1.19–7.8; *P *< 0.001). However, the separate analysis reduced the significance of the reported association for *BRCA1* mutation carriers. 32 of the cases, who were defined by history of IVF, and 73 of the controls reported use of HRT, while 136 of the cases and 800 of the controls declared no use. Surveillance of cases and controls ended at RRBSO, which was performed on a total of 114 participants. All of the participants were Jewish Israeli women with personal or family history of BRCA mutation-associated cancers, leading to an overrepresentation of a specific ethnic group. Numbers of cases and controls who used HRT were low and there was no information provided on duration of use and different formulations.

### Breast cancer

Regarding breast cancer, we found one study on the association between HRT and risk of cancer in mutation carriers with and without RRBSO. All of them included *BRCA1* mutation carriers, only. Furthermore, we identified data from five publications on breast cancer risk associated with HRT use in *BRCA1/2* mutation carriers undergoing RRBSO (Table 2).

**Table 2:** HRT and risk of breast cancer in BRCA mutation carriers

Study/study design/Oxford center of evidence based medicine (OCEBM) level of evidence (LOE)	Number	Results
*BRCA1 mutation carriers with and without RRBSO*		
Eisen et al. (2008) Case–control study LOE 3b	236 case–control pairs *BRCA1* 236 case–control pairs *BRCA1*	Decrease in risk OR = 0.58; 95% CI 0.35–0.96; *P *= *0*.03 Significant risk reduction associated with ET (OR = 0.51; 95% CI 0.27–0.98; *P *= 0.04), non-significant risk reduction associated with EPT (OR = 0.66; 95% CI 0.34–1.27; *P *= 0.21)
Kotsopoulos et al. (2016) Case–control study LOE 3b *Extension study to *Eisen et al. (2008)	432 case–control pairs *BRCA1*	No increase in risk OR = 0.80; 95% CI 0.55–1.16; *P *= 0.24 No association with duration of use
*BRCA1/2 mutation carriers after RRBSO*		
Rebbeck et al. (1999) Case–control study LOE 3b	122 *BRCA1 (*43 cases with RRBSO, 79 controls without surgery)	Decrease in breast cancer risk associated with RRBSO after exclusion of HRT users HR = 0.42; 95% CI = 0.22–0.81 No negation of risk reduction associated with RRBSO
Armstrong et al. (2004) Markov Chain decision model LOE 3b	Computer based model	Loss of life expectancy when HRT (EPT) continued for life
Rebbeck et al. (2005) Prospective cohort study LOE 1b	315 *BRCA1* (110 with RRBSO), 147 *BRCA2* (45 with RRBSO)	HRT without impact on risk reduction associated with BSO HR = 0.37; 95% CI 0.14–0.96 No association with different types of HRT
Domchek et al. (2011) *Extension and follow-up to* Rebbeck et al. (2005)	795 *BRCA1,* 504 *BRCA2* (321 with RRBSO)	No increase in risk overall *BRCA1*: decrease in risk for women with (HR = 0.52; 95% CI 0.30–0.92) and without RRBSO (HR = 0.29; 95% CI 0.13–0.69) No association with different types of HRT
Kotsopoulos et al. (2018) Prospective cohort study LOE 1b	872 *BRCA1* (all with RRBSO)	No increase in risk overall HR = 0.97; 95% CI 0.62–1.52; *P *= 0.89 Possible adverse effect of progestin-based HRT
Marchetti et al. (2018) Metaanalysis LOE 2a Included studies: *(a) Two prospective cohort studies (LOE 1b): *Rebbeck et al. (2005), Kotsopoulos et al. (2018) *(b) One retrospective cohort study (LOE 3b): *Gabriel et al. (2009)	a) Rebbeck et al. (2005): *462 BRCA1/2 (155 with RRBSO)* Kotsopoulos et al. (2018): 872 *BRCA1 (all with RRBSO)* b) Gabriel et al. (2009): 47 *BRCA1,* 26 *BRCA2*	No increase in risk for the entire cohort (HR = 1.01; 95% CI 0.16–1.54) No increase in risk for prospective trials in separate analysis (HR = 0.98; 95% CI 0.63–1.52)

#### BRCA1 mutation carriers with and without RRBSO

Kotsopoulos et al. included 432 matched pairs with *BRCA1* mutation in their case–control study from 2016 (Kotsopoulos et al. 2016). A number of the included participants overlap with a prospective cohort study conducted by Rebbeck et al. in 2005, which will be discussed below (Rebbeck et al. 2005).

Also, the case–control study by Kotsopoulos et al. is an extension to an earlier report by Eisen et al. published in 2008, which included 236 matched case–control pairs with *BRCA1* mutation (Eisen et al. 2008). For ever compared to never use of HRT, a decrease in breast cancer risk was observed (OR = 0.58; 95% CI 0.35–0.96; *P *= 0.03). When different formulations of HRT were examined, Eisen et al. found a significant risk reduction associated with use of ET (OR = 0.51; 95% CI 0.27–0.98; *P *= 0.04) and a non-significant risk reduction associated with EPT (OR = 0.66; 95% CI 0.34–1.27; *P *= 0.21). Information on estrogen receptor (ER) status was available in 103 of the cases (44%). In 12% of the cases with ER-positive and 23% with ER-negative breast cancer, HRT was reported. Eisen et al. concluded that HRT might not be contraindicated for *BRCA1* mutation carriers.

However, multivariate analysis in the extension study by Kotsopoulos et al. found no association between breast cancer risk and ever compared to never use of HRT (OR = 0.80; 95% CI 0.55–1.16; *P *= 0.24). Also, no association with different HRT formulations was observed (*P *≥ 0.11). 46 of the cases and 42 of the controls reported ET use. 28 of the cases and 41 of the controls used EPT, while 2 cases and 3 controls reported progesterone monotherapy.

The type of menopause was natural in 327 (75.7%) of the cases and controls, and surgical in the remaining participants. Women after bilateral mastectomy were excluded from the study. The mean age at enrollment was 58 years for cases and 58.3 for controls. 18.5% of the cases and 21.3% of the controls reported ever use of HRT (*P *= 0.31). The mean duration of use was 4.42 years for cases and 4.27 for controls (*P *= 0.83). Analysis was carried out separately for surgical and natural menopause. The odds ratio for women with surgical menopause was higher (OR = 1.06; 95% CI 0.58–1.96; *P *= 0.85) than for those with natural menopause (OR 0.72; 95% CI 0.44–1.18; *P *= 0.20), although the difference was not statistically significant. The results did not differ by duration (≥ 3 years and ˂3 years) or recency of use. Furthermore, no association was observed with age at menopause (≤ 45 years of age or more) and breast cancer diagnosis (≤ 50 years of age or more).

#### BRCA1/2 mutation carriers after RRBSO

Rebbeck et al. included a total of 122 BRCA1 mutation carriers in their matched case––control study from 1999 (Rebbeck et al. 1999). They included 43 healthy carriers with RRBSO and 79 controls without surgery to examine the breast cancer risk after RRBSO. The study found a significant reduction in breast cancer risk associated with RRBSO in the total sample (HR = 0.53; 95% CI 0.33–0.84). When women with HRT exposure were excluded from the analysis (*n* = 22), a further decrease in risk was observed (HR = 0.42; 95% CI = 0.22–0.81). As the HR estimate in the total sample was only marginally higher, the authors assumed no more than a moderate increase in breast cancer risk associated with HRT after prophylactic surgery. They conclude, that HRT use does not negate the reduction in breast cancer risk associated with RRBSO. The mean age of the healthy carriers at the time of surgery was 39.4 years, the mean corresponding age of the controls was 35.3. Women who had undergone mastectomy prior to BSO were excluded. The cases were followed up for an average of 9.6 years after surgery, the controls for an average of 8.1 years. 69% of the cases and 6% of the controls reported ever use of HRT. The sample size of the study was small and there was no complete information on the menopausal status of the participants. Information on timing, duration, dose, and different formulations of HRT was not provided and data on HRT use were not complete.

Armstrong et al. used a Markov decision analytic model to investigate the impact of HRT after RRBSO in premenopausal *BRCA1/2* mutation carriers (Armstrong et al. 2004). HRT was defined as combined EPT. A gain in life expectancy associated with RRBSO between the age of 30 and 40 years was found, irrespective of whether or not HRT was used. The decrease of ovarian and cancer risk outweighed the increase in risk of cardiovascular diseases and osteoporosis. Additional prophylactic bilateral mastectomy was associated with a further increase in life expectancy.

For *BRCA1/2* mutation carriers without bilateral mastectomy, HRT from the time of RRBSO until the age of 50 years was associated with relatively small changes in life expectancy (ranging from − 0.34 years for 30-year-old women to + 0.17 years for 40-year-old women). When HRT was continued for life, a decrease in life expectancy was observed (ranging from − 1.09 to − 0.76 years, respectively).

Use of HRT until the age of 50 years after bilateral mastectomy and oophorectomy resulted in a gain in life expectancy (ranging from 0.78 years for 30-year-old women to 0.79 years for 40-year-old women). When HRT was continued for life, the gain in life expectancy in this subgroup dropped to an amount of 0.39 and 0.37 years.

The PROSE study group around Rebbeck et al. included 462 *BRCA1/2* mutation carriers for a prospective cohort study in 2005 (Rebbeck et al. 2005). The authors report a partial overlap with their publication from 1999, which has been discussed previously (Rebbeck et al. 1999). However, the authors emphasize the recruitment of additional participants and different inclusion and exclusion criteria for the two studies. In the study by Rebbeck et al., HRT exposure had no impact on the reduction of breast cancer risk associated with RRBSO in *BRCA1/2* mutation carriers (HR = 0.37; 95% CI 0.14–0.96) compared to the entire cohort. 155 of the included women underwent RRBSO. The majority, 71% of the included women with and 67% of those without RRBSO, had a *BRCA1* mutation. Bilateral mastectomy led to an exclusion from the study, as well as a history of ovarian or breast cancer before or within 6 months after recruitment. 12 of the *BRCA1/2* mutation carriers with and 65 without RRBSO were diagnosed with breast cancer during follow-up. Follow-up was 3.6 years after RRBSO. The mean age at RRBSO was 42.7 years. The mean age at breast cancer diagnosis was 45.6 for the cases with and 39.3 for the controls without RRBSO. Different formulations of HRT were also examined. No difference was found, although the sample sizes were relatively small with 54 women who took ET and 34 who took EPT, respectively. Separate analyses for *BRCA1* and BRCA2 mutation carriers were not conducted and an association with the duration of use was not examined.

In 2011, Domchek et al. published data on an extension and follow-up to this study (Domchek et al. 2011). In total, 795 *BRCA1* and 504 *BRCA2* mutation carriers were included. 321 women underwent RRBSO, of whom 45% used HRT after surgery. Mean follow-up was 5.4 years after RRBSO. Like in the initial study, HRT following RRBSO was not associated with an increase in breast cancer risk. When different types of HRT were examined, neither ET nor EPT led to an increase in risk. For *BRCA1* mutation carriers, a decrease in breast cancer risk associated with HRT was found for both women with (HR = 0.52; 95% CI 0.30–0.92) and without RRBSO (HR = 0.29; 95% CI 0.13–0.69).

Kotsopoulos et al. conducted a prospective cohort study in 2018 which included 872 *BRCA1* mutation carriers undergoing RRBSO (Kotsopoulos et al. 2018). A subset overlapped with the previously performed case–control study by the same authors (Kotsopoulos et al. 2016). The primary endpoint was incident invasive breast cancer, which was diagnosed in 92 (10.6%) of the included carriers. Women with a prior diagnosis of breast cancer or with a history of bilateral mastectomy were excluded from the study. The mean range between RRBSO and breast cancer diagnosis was 4.5 years.

The results did not show an association between breast cancer risk and HRT after RRBSO (HR = 0.97; 95% CI 0.62–1.52; *P *= 0.89). However, differing effects associated with different types of HRT were observed, suggesting a possible adverse effect of progestin-based HRT. 377 (43%) women used HRT after oophorectomy and 495 (57%) did not. The mean duration of HRT use was 3.9 years. Women who used HRT had a mean age of 40.3 years at enrollment, women who did not a mean age of 45.8. The mean age at RRBSO was 43.0 and 48.4 years, respectively (*P *< 0.0001). Mean follow-up duration was 7.9 years for women who used HRT and 7.4 years for women who did not (*P *< 0.07). Among the HRT users, 259 (69%) used ET, 66 (18%) EPT, and 40 (11%) used progesterone monotherapy. The remaining 21% used other formulations. After 10 years of follow-up, the cumulative incidence of breast cancer was significantly lower for *BRCA1/2* mutation carriers who used ET (12%) compared to those who used EPT (22%; *P *= 0.04). The observed effect was even more pronounced for women prior to the age of 45 years (9% and 24%, respectively; *P *= 0.009).

Marchetti et al. included two prospective and one retrospective cohort studies in their metaanalysis published in 2018 (Marchetti et al. 2018). In total, the three studies comprised 1100 *BRCA1/2* mutation carriers after RRBSO (Rebbeck et al. 2005; Kotsopoulos et al. 2018; Gabriel et al. 2009).

The two prospective cohort studies by Rebbeck et al. and Kotsopoulos et al. have already been described above (Rebbeck et al. 2005; Kotsopoulos et al. 2018). In the retrospective cohort study by Gabriel et al. 47 *BRCA1* and 26 *BRCA2* mutation carriers were included (Gabriel et al. 2009). The main aim of this study was to investigate differences in HRT use associated with and without total abdominal hysterectomy at the time of RRBSO in *BRCA1/2* mutation carriers. History of breast cancer led to exclusion of the study. 55% of the included women underwent hysterectomy in addition to RRBSO. 17 of the hysterectomized and 16 of the non-hysterectomized women used HRT and 50% of the HRT users had a prophylactic mastectomy. 3 of 17 women who used ET and 9 of 29 women who did not use HRT developed breast cancer. Although HRT use was not associated with more breast cancer cases, no data on estimated breast cancer risk were given and no further analysis was performed.

In the metaanalysis, no increase in breast cancer risk associated with HRT was found for the entire cohort (HR = 1.01; 95% CI 0.16–1.54). Metaanalysis was also performed separately for the two prospective trials, but no negative impact on breast cancer risk was found (HR = 0.98; 95% CI 0.63–1.52). The mean duration of HRT was 3.3 years. Different HRT formulations were also examined. 326 of included women used ET and 114 used EPT. No increase in risk was found associated with the different types of HRT. However, breast cancer risk associated with ET use was lower compared to use of EPT, both in the entire cohort (OR = 0.62; 95% CI 0.29–1.31) and in the prospective studies only (OR = 0.62; 95% CI 0.29–1.31).

Marchetti et al. conclude, that HRT following RRBSO in *BRCA1/2* mutation carriers seems to be a safe therapeutic option. However, they point out the low number especially of *BRCA2* mutation carriers and the need of larger prospective and randomized studies in the future.

### Endometrial cancer

Segev et al. included 62 cases and 951 controls with *BRCA1* and 21 cases and 76 controls with *BRCA2* mutation in their case–control study (Segev et al. 2015) (Table 3). No overall association between HRT and endometrial cancer was found (OR = 0.73; 95% CI 0.33–1.63; *P *= 0.44). However, an increase in risk for endometrial cancer associated with progestin-treatment (OR = 6.91; 95% CI 0.99–98.1; *P *= 0.05) and a non-significant decrease in risk associated with ET (OR = 0.23; 95% CI 0.03–1.78; *P *= 0.16) were observed. The separate analysis for *BRCA1* and *BRCA2* mutation carriers did not show an overall association between HRT and endometrial cancer, either. For *BRCA1* mutation carriers, multivariate analysis showed a non-significant risk reduction associated with ET (OR = 0.52; 95% CI 0.06–4.23; *P *= 0.54) a non-significant increase in risk associated with EPT (OR = 1.77; 95% CI 0.20–2.98; *P *= 0.70). Progestin-only HRT was associated with a significant increase in endometrial cancer risk (OR = 16.5; 95% CI 2.02–134 *P *= 0.009) in BRCA1 mutation carriers. In *BRCA2* mutation carriers, EPT was associated with a decrease in risk (OR = 0.35; 95% CI 0.02–5.15; *P *= 0.44). Due to small numbers, no analyses could be conducted for ET and progestin therapy in *BRCA2* mutation carriers. The mean age at baseline was 55.5 years for cases and 56.1 for controls (*P *= 0.65). 28.9% of the cases and 41.9% of the controls were premenopausal, compared to 71.1% and 58.1% who were postmenopausal (*P *= 0.02). 13 (16.1%) of the cases and 157 (15.9%) the controls reported use of HRT (*P *= 0.96). 2 of the cases and 42 of the controls took ET, 8 and 63 took EPT, respectively. 1 of the cases and 48 of the controls reported use of other formulations or type of HRT was unknown. Numbers and exposure to HRT were small: only 13 of the cases used HRT, 2 of which took ET. No information was given on duration and timing of HRT.

**Table 3:** HRT and risk of endometrial cancer in BRCA mutation carriers

Study/study design/Oxford Center of Evidence based Medicine (OCEBM) level of evidence (LOE)	Number	Results
Segev et al. (2015) Case–control study LOE 3b	Cases: 62 *BRCA1*, 21 *BRCA2* Controls: 951 *BRCA1*, 76 *BRCA1*	No increase in risk overall OR = 0.73; 95% CI 0.33–1.63; *P *= 0.44 Increase in risk associated with progesterone-only (OR = 6.91; 95% CI 0.99–98.1; *P *= 0.05), non-significant decrease in risk associated with estrogen-only (OR = 0.23; 95% CI 0.03–1.78; *P *= 0.16)

## Discussion

For ovarian cancer, one of the studies showed no overall association between HRT and cancer risk in *BRCA1/2* mutation carriers (Kotsopoulos et al. 2006). However, an increase in risk associated with estrogen- and a decrease in risk associated with progestin-containing HRT was observed, each being non-significant. The second study which primarily investigated the effect of fertility treatments on ovarian cancer reported an increase in risk associated with HRT (Perri et al. 2015). However, numbers of women in this study were low and data on different formulations of HRT were lacking.

For breast cancer, most of the studies including *BRCA1* mutation carriers with and without RRBSO did not find an increase in risk associated with HRT, neither for woman after surgical, nor after natural menopause (Kotsopoulos et al. 2016; Eisen et al. 2008). One of the studies even found a significant reduction of breast cancer risk associated with ever use of HRT as well as with ET and a non-significant risk reduction associated with EPT (Eisen et al. 2008). However, the later extension to this study by Kotsopoulos et al. did not confirm the finding of a reduction in risk (Kotsopoulos et al. 2016).

The studies investigating an association between HRT and breast cancer risk in *BRCA1/2* mutation carriers after RRBSO mostly did not find an adverse effect, either (Rebbeck et al. 2005; Kotsopoulos et al. 2018; Marchetti et al. 2018). One case–control study by Rebbeck et al. could not exclude a moderate increase in breast cancer risk associated with HRT, although the authors concluded that the reduction in breast cancer risk associated with RRBSO was not abolished by the use of HRT (Rebbeck et al. 1999). A subsequent prospective study by the same authors found no impact on the reduction of breast cancer risk associated with RRBSO in *BRCA1/2* mutation carriers (Rebbeck et al. 2005). The extension and follow-up data from 2011 even reported a decrease in breast cancer risk associated with HRT in *BRCA1* mutation carriers with and without RRBSO (Domchek et al. 2011). In contrast, Armstrong et al. postulated an adverse effect on life expectancy in their Markov Chain decision model, when HRT was continued for life after risk-reducing surgery (Armstrong et al. 2004). However, HRT was defined as EPT and no other formulations were examined. No relevant effect was found when HRT was stopped at the age of 50 years. The analytic model was based on analyses of the effects of HRT among women with a family history of breast cancer, not specifically on *BRCA1/2* mutation carriers, where HRT might have a different effect. Also, the analytic model from 2004 might not contain current assumptions and may be outdated. Therefore, it might not be appropriate for current clinical decision making.

Regarding different types of HRT, the prospective study by Rebbeck et al. as well as its extension by Domcheck et al. did not find an association with breast cancer risk (Rebbeck et al. 2005; Domchek et al. 2011). Another prospective cohort study by Kotsopoulos et al. observed a significantly lower cumulative incidence of breast cancer in *BRCA1/2* mutation carriers who used ET compared to EPT after RRBSO after 10 years of follow-up, suggesting a possible adverse effect of progestin-based HRT (Kotsopoulos et al. 2018). These two study groups were the first to contribute prospective data to the discussion about HRT in *BRCA* mutation carriers, focusing exclusively on mutations in *BRCA1*. In the metaanalysis by Marchetti et al., another smaller retrospective study was additionally included and a lower breast cancer risk associated with the use of ET compared to EPT was observed (Marchetti et al. 2018). However, no actual increase in risk associated with the different types of HRT was found.

Recently, the safety of endocrine interventions in BRCA1/2 mutation carriers has gradually gained attention. A number of systematic reviews addressing breast cancer risk associated with HRT following RRBSO in *BRCA1/2* mutation carriers have been published (Siyam et al. 2017; Birrer et al. 2018; Gordhandas et al. 2019; Vermeulen et al. 2019; Gaba and Manchanda 2020). According to one of those reviews, the safety of HRT is demonstrated best in the PROSE study by Rebbeck in 2005 (Birrer et al. 2018). In contrast, another review points out the small number of women who took EPT in this study and concludes that safety of HRT types other than ET cannot be definitely evaluated based on these data (Gordhandas et al. 2019). In general, most of the reviewers conclude, that there is no evidence for an increase in breast cancer risk in *BRCA1/2* mutation carriers after RRBSO, but that more data are needed to draw clear conclusions. Vermeulen et al. especially point out the possible adverse effect of progestin-containing HRT found by Kotsopoulos et al. and the need of further investigation in this direction (Kotsopoulos et al. 2018; Vermeulen et al. 2019). In contrast, the recently published review by Gaba et al. did not find a change in clinical practice justifiable based on this study due to small numbers and inconsistent follow-up (Gaba and Manchanda 2020). All of the reviews explicitly investigated the safety of HRT in *BRCA1/2* mutation carriers after RRBSO. However, most of them also presented studies which included women with and without RRBSO (Birrer et al. 2018; Gordhandas et al. 2019; Gaba and Manchanda 2020). A clear separation and differentiated evaluation between these two subgroups was lacking.

Regarding endometrial cancer risk associated with HRT use in *BRCA1/2* mutation carriers, we found only one publication (Segev et al. 2015). In contrast to the general population, an increase in risk associated with progesterone monotherapy and a non-significant decrease in risk associated with estrogen monotherapy was observed. When the analysis was performed separately for *BRCA1*, the described effect was confirmed and a non-significant increase in risk associated with EPT was seen. In contrast, a decrease in risk associated with EPT was observed in *BRCA2* mutation carriers. However, numbers especially of cancer cases and exposure to HRT were small. There were only two *BRCA1* mutation carriers diagnosed with endometrial cancer who were exposed to ET and analyses associated with monotherapy could not be performed in *BRCA2* mutation.

Overall, data on cancer risk associated with HRT use in *BRCA1/2* mutation carriers are limited. Sample sizes were often small, especially regarding *BRCA2* mutation carriers which were included in only few studies (Kotsopoulos et al. 2006; Perri et al. 2015; Rebbeck et al. 2005; Gabriel et al. 2009). In addition, the majority of these studies did not perform subgroup analyses for *BRCA1* and *BRCA2* (Kotsopoulos et al. 2006; Rebbeck et al. 2005; Gabriel et al. 2009). Between some of the studies regarding breast cancer risk, a partial overlap was reported (Kotsopoulos et al. 2016; Rebbeck et al. 2005; Eisen et al. 2008; Domchek et al. 2011). Although most of the included studies in this review examined the impact of different HRT formulations, none of them took different dosages or treatment routines, such as sequential or continuous combined HRT, into consideration.

Due to elevated risk of cancer, *BRCA1/2* mutation carriers are increasingly encouraged to undergo RRBSO after completion of childbearing (Birrer et al. 2018; Domchek and Kaunitz 2016). However, there are studies suggesting that early menopause and subsequent estrogen loss does not only cause a decrease in quality of life due to menopausal symptoms, but may also increase cardiovascular diseases, osteoporosis, and cognitive dysfunction (Parker et al. 2009; Georgakis et al. 2019). Also, there are indications that BSO is associated with an increased mortality in the general population, especially when BSO is performed at a younger age of 45–50 years and no HRT is used (Parker et al. 2009; Rocca et al. 2006). In contrast, large prospective studies observed a decrease in all-cause mortality associated with RRBSO in *BRCA1/2* mutation carriers compared to no RRBSO. If HRT until the age of natural menopause could increase survival further has not been analyzed so far. However, there are data supporting an effect of HRT after RRBSO in *BRCA1/*2 mutation carriers on menopausal symptoms such as hot flashes (Madalinska et al. 2006). Although the Women´s Health Initiative, the largest study to date on peri- and postmenopausal HRT, did not find a protective cardiovascular effect associated with HRT, younger women might still benefit (Rossouw et al. 2007; Manson et al. 2007).

Although data are limited, HRT does not seem to be associated with relevant increases in breast cancer risk according to the available studies, especially for *BRCA1/2* mutation carriers who had RRBSO. Given the risks of RRBSO in younger premenopausal women, HRT can, therefore, be recommended after RRBSO prior to the age of 50 years. ET is associated with an increasing risk of endometrial cancer and, therefore, not recommended for non-hysterectomized women. But also long-term EPT can cause an increase in endometrial cancer risk. (Razavi et al. 2010) Risk for endometrial cancer in *BRCA1/2* mutation carriers is being discussed controversially. There are some studies which suggest a higher risk of endometrial cancer in *BRCA1/2* mutation carriers (Laitman et al. 2019). Segev et al. found a higher risk of endometrial cancer among *BRCA1* mutation carriers compared to the general population which, however, was largely attributable to the use of Tamoxifen (Segev et al. 2013). According to existing data, mutation in *BRCA1/2* genes alone is not an indication for hysterectomy. Hysterectomy at the time of RRBSO, therefore, needs to be discussed individually depending on age and additional risk factors.

In conclusion, there is insufficient evidence on the effect of HRT on ovarian and endometrial cancer in *BRCA1/2* mutation carriers. Also, data on the association of HRT use and breast cancer in *BRCA1/2* mutation carriers are limited, especially for *BRCA2* mutation carriers. More prospective studies with longer follow-up periods are needed to assess the safety and to further investigate different formulations, doses and regimen of HRT. Due to elevated cancer risk, RRBSO is recommended at the age of about 35–40 years in *BRCA1* and 40–45 years in *BRCA2* mutation carriers, whereby family planning and the earliest age at diagnosis in affected family members have to be taken into consideration (Wagner and Reuß 2019; The Australian Cancer Network and National Breast Cancer Centre 2004). However, as ovarian cancer can occur also before the age of 35 years and data on RRBSO in *BRCA1/2* mutation carriers is still limited, the ideal timing of RRBSO remains difficult (Finch et al. 2014; Marchetti et al. 2014). According to limited data from available studies, HRT following RRBSO in *BRCA1/2* mutation carriers does not seem to have an adverse effect or negate the reduction in breast cancer risk associated with RRBSO. Adequate HRT after RRBSO should be offered to premenopausal women to avoid postmenopausal symptoms and chronic diseases resulting from low estrogen levels such as osteoporosis and myocardial infarction. Regarding *BRCA1/2* mutation carriers who have not undergone RRBSO, HRT does not seem to have a relevant impact on cancer risk. Short-term HRT may be considered in *BRCA1/2* mutation carriers without RRBSO to reduce perimenopausal symptoms. However, indication should be strict and patients should be informed about limited data on the safety concerning cancer risk. ET is recommended after hysterectomy but should be avoided in non-hysterectomized women due to possible increases in endometrial cancer risk.
